# Preparation of Pebax 1657/MAF-7 Mixed Matrix Membranes with Enhanced CO_2_/N_2_ Separation by Active Site of Triazole Ligand

**DOI:** 10.3390/membranes12080786

**Published:** 2022-08-16

**Authors:** Xingqian Wang, Yuping Zhang, Xinwei Chen, Yifei Wang, Mingliang He, Yongjiang Shan, Yuqin Li, Fei Zhang, Xiangshu Chen, Hidetoshi Kita

**Affiliations:** 1 State-Province Joint Engineering Laboratory of Zeolite Membrane Materials, Institute of Advanced Materials (IAM), College of Chemistry and Chemical Engineering, Jiangxi Normal University, Nanchang 330022, China; 2 The Attached Middle School to Jiangxi Normal University, Nanchang 330031, China; 3 Environmental Science and Engineering, Graduate School of Science and Engineering, Yamaguchi University, Ube 755-8611, Japan

**Keywords:** Pebax 1657/MAF-7 mixed matrix membranes, active site of triazole ligand, CO_2_/N_2_ separation, high CO_2_/N_2_ selectivity

## Abstract

Fillers play a critical role in the performance of mixed matrix membranes (MMMs). Microporous metal azolate frameworks (MAFs) are a subclass material of metal–organic frameworks (MOFs). Due to the uncoordinated nitrogen of the organic ligands, MAF-7 (SOD-[Zn(mtz)_2_], Hmtz = 3-methyl-1,2,4-triazole, window: d = 0.34 nm) shows excellent CO_2_ adsorption performance. In this work, Pebax 1657/MAF-7 MMMs were prepared by a sample solution casting method with MAF-7 particles as fillers for the first time. By means of X-ray diffraction (XRD), scanning electron microscope (SEM), infrared radiation (IR), and thermogravimetry (TG), the compositional and structural properties of the mixed matrix membrane with different filler content were analyzed. The results show that the compatibility of MAF-7 and Pebax is good with a filler content of 5 wt.%. The pure gas testing showed that mixed matrix membrane has a high ideal CO_2_/N_2_ selectivity of 124.84 together with a better CO_2_ permeability of 76.15 Barrer with the optimized filler content of 5 wt.%. The obtained membrane showed 323.04% enhancement in selectivity of CO_2_/N_2_ and 27.74% increase in the permeability of CO_2_ compared to the pristine membrane at 25 °C and 3 bar. The excellent separation performance may be due to the ligands that can afford a Lewis base active site for CO_2_ binding with the uniform dispersion of MAF-7 particles in Pebax and the favorable interface compatibility. The obtained membrane overcomes the Robeson’s upper bound in 2008 for CO_2_/N_2_ separation. This work provides a new strategy by utilizing MAFs as fillers with triazole ligand to enhance the gas separation performance of mixed matrix membranes.

## 1. Introduction

With the growing demand for energy in the modern society, the excessive emission of CO_2_ from fossil fuel combustion has been causing environmental problems. Hence, the CO_2_ capture and storage has attracted more and more attention in the past decades [1,2]. Compared with conventional separation such as adsorption and cryogenic purification, membrane separation technology plays an important role in the field of gas separation with the advantages of low energy consumption, low running costs, and environmental friendliness [3,4]. Many kinds of polymers have been adopted for preparing polymer membrane, such as cellulose acetate (CA), polydimethylsiloxane (PDMS), and polyethylene oxide (PEO). However, polymer membranes often suffer from the Robeson upper bound that is the trade-off between selectivity and permeability [5,6]. Therefore, it is very important to develop new membrane materials to break the limit through tuning transmission path and/or adsorption site. Mixed matrix membranes (MMMs), as a composite membrane, are assembled by introducing inorganic fillers into a continuous polymer phase [7,8]. It brings together the advantages of inorganic materials and polymers and has been rapidly developed in the past two decades [9,10,11,12,13,14]. For the inorganic phase, many porous materials have been incorporated into the polymer matrix to construct MMMs, such as zeolite [15,16,17], graphene oxide (GO) [18,19], carbon nano tubes (CNTs) [20,21,22,23], etc. However, inorganic fillers have poor compatibility with the polymer phase in the MMMs, which always lead to the obtained membrane showing a poor selectivity. To increase the compatibility between the inorganic and organic phases, currently, various modification techniques for improving the compatibility between the polymers and the particles have been reported [13,24,25,26]. As an emerging porous material, MOFs are mainly composed of metal clusters and organic ligands [27] and have great potential in the field of gas separation. They have many advantages, such as strong adsorption capacity, orderly pore structure, and adjustable surface properties [15,28]. Due to the good mutual compatibility, there are many reports about the MOF-based mixed matrix membrane, such as UiO-66 [24], ZIF-301 [29], UiO-67 [30], MIL-53 [26,31], and MOF-801 [32], etc. In addition, enhancing chemical affinity towards guest molecules is an effective way to improve the gas separation performance of the membrane. Casado-Coterillo et al. [33] utilized ((emim)(Ac)) IL to improve the compatibility of ZIF-8-Chitosan interface in order to enhance the CO_2_/N_2_ separation performance of their synthesized MMMs. Jin et al. [24] reported UiO-66 and UiO-66-NH_2_ nanocrystals as additive and incorporated these particles in Pebax-1657, the results showed much greater CO_2_ permeability of UiO-66-NH_2_ than that of UiO-66 in MMMs, due to the CO_2_-philic nature of UiO-66-NH_2_ particles. The PSf/ZIF-8/NH_2_ showed superior CO_2_/CH_4_ selectivity due to the presence of the CO_2_-philic group, amine [34]. Although the above membranes showed comparable performance, the chemical modification process of fillers was very complex, and the fillers with functional groups were easily destroyed during the membrane preparation process. So far, it is still necessary to find a suitable filler with affinity toward guest molecules, accompanying a nice compatibility with polymers.

MAF-7 has the same SOD topological structure as ZIF-8 (also named MAF-4), which is one of the most studied MOF materials [35,36,37]. The triazolate ligand in MAF-7 structure uses only the 2- and 4-N atoms for coordination, leaving the 1-N atom unbound [38]. Leading to the ligand being able to provide a Lewis base active site for guest binding. Hence, the isosteric heats for MAF-7 (23.8–25.1 kJ mol^−1^) are obviously higher than those for ZIF-8 (14.9–17.2 kJ mol^−1^). Therefore, MAF-7 may be an ideal candidate filler in preparing mixed matrix membranes for CO_2_ separation due to its enhanced CO_2_ adsorption capacity.

For another component in mixed matrix membranes, organic phase also plays a vital role in gas separation. Glassy polymers have superior solubility selectivity due to their symmetric polymer chain’s structure, but plasticization at higher operating pressures lead to the membrane showing a low selectivity. However, rubbery polymers, due to their amorphous structures, are less prone to plasticization effects. As a rubbery polymer, the commercialized block copolymer Pebax-1657 is composed of two parts: 60% polyethylene oxide (PEO) section and 40% polyamide (PA) section. The PEO section can provide strong affinity with CO_2_ that has quadrupole–dipole interaction with PEO, while the PA section provides mechanical strength [39].

Based on the high CO_2_ adsorption capacity of Pebax 1657 and MAF-7, Pebax 1657/MAF-7 MMMs may show high separation performance for CO_2_/N_2_. Herein, our purpose was to construct Pebax-1657/MAF-7 MMMs for the first time by a simple solution casting method. The as-synthesized MAF-7 crystals were used as fillers, different from the fillers which were obtained by complicated chemical modification methods, the as-synthesized MAF-7 fillers maintain a good affinity with CO_2_ molecule due to the uncoordinated N-donor existing in its ligand structure; hence, the sophisticated modification reaction process of fillers during membrane synthesis can be avoided. The effect of MAF-7 loading, testing temperature, and feed pressure on the permeability and selectivity were systematically investigated. The SEM images showed that the MAF-7 particles were homogeneously dispersed in the organic phase, and there were no obvious interface defects between the fillers and the Pebax, combining the uncoordinated nitrogen of the organic ligands, the resulting MMM shows excellent CO_2_/N_2_ selectivity. This work provides a strategy by utilizing MAFs as fillers with triazole ligand to enhance the gas separation performance of mixed matrix membranes.

## 2. Materials and Methods

### 2.1. Materials

Commercial Pebax-1657 (60 wt.% polyethylene oxide (PEO) and 40 wt.% polyamide (PA)) was supplied by Arkema (Colombes, France). Polyvinylpyrrolidone (PVP, average molecular weight 10000, white powder) was purchased from Aladdin Chemical Co., Ltd. (Shanghai, China). Ammonia water (25%) was purchased from Tianjin Zhiyuan Chemical Reagent Co., Ltd. (Tianjin, China). Methanol (≥99.5%) was purchased from Tianjin Zhiyuan Chemical Reagent Co., Ltd. (Tianjin, China). Zn(NO_3_)_2_·6H_2_O (≥99.0%) was obtained from Fortune Chemical Reagent Co., Ltd. (Suzhou, China). The 3-methyl-1,2,4-triazole (Hmtz) (98.2%, white powder) was purchased from Aladdin Chemical Co., Ltd. (Shanghai, China). N_2_ and CO_2_ (purity: 99.6%) were purchased from Jiangxi Huadong Special Gas Co., Ltd. (Nanchang, China). All these raw materials and solvents were used without further purification. Deionized water was homemade in our laboratory.

### 2.2. Synthesis of MAF-7 Particles

MAF-7 particles were fabricated based on the reference with a slight modification [38]. In detail, first, 0.672 g Hmtz and 0.297 g Zn(NO_3_)_2_·6H_2_O were dissolved in two vials containing 5 mL deionized water, respectively. Second, the vials were stirred continuously for about 10 min followed by adding 1 mL ammonia to the zinc source solution and stirred for 5 min. Third, the above solutions were mixed and 0.100 g PVP was added into the solution with stirring for 10 min. Last, the solution above was transferred into the autoclave and put it into the oven at 120 °C for 4 h. The obtained crystals were washed with methanol by centrifugation three times. The MAF-7 powders were dried and activated in a 60 °C vacuum oven overnight.

### 2.3. Preparation of Pebax 1657/MAF-7 Mixed Matrix Membranes

The mixed matrix membranes based on MAF-7 crystals were made by the solution casting method. The prepared MAF-7 powders with different loading of 0 wt.%, 1 wt.%, 3 wt.%, 5 wt.%, 7 wt.%, and 9 wt.% were uniformly dispersed in the mixed solvent of ethanol and deionized water (mass ratio 70/30) under continuous reflux for 0.5 h at 80 °C. Then the suspension was stirred for 5 min, and ultrasounded for 1 h. Half of the Pebax pellets were added into the mixed solution and refluxed to induce dissolution, then the rest of the polymers were added into the flask and sonicated for another 1 h. Next, the flask was stirred in an oil bath under reflux at 80 °C for 24 h to obtain a uniform mixed Pebax/MAF-7 membrane solution. A stainless-steel ring was fixed on a glass pane to construct MMMs. The casting solution was added by a pipette into the stainless-steel ring in the clean room, then the membrane was transferred to the 60 °C vacuum oven for drying overnight, peeled off carefully from the substrate, and stored in a desiccator. Based on the MOF loading, the fabricated membrane abbreviations are given in Table 1.

Filler loading is calculated as follows:

(1) $$\mathrm{Filler}\;\mathrm{loading}\;\left(\mathrm{wt}.\%\right)=\frac{M_{filler}}{M_{filler}+M_{polymer}}*100\%$$

### 2.4. Characterization Techniques

The crystalline phase of MAF-7 powders, pure membrane, and Pebax 1657/MAF-7 mixed matrix membranes were confirmed by X-ray diffraction (XRD, Ultima IV, Rigaku, Tokyo, Japan) with Cu-Kα (λ = 1.54 Å) radiation in the 2θ range between 5° and 45°. The morphology and size of the above samples were observed by field emission scanning electron microscope (SEM, SU8020, Hitachi, Tokyo, Japan), with an accelerating voltage of 5 kV. The MMM samples were prepared by freeze cutting under liquid nitrogen followed by a sputter coating of the gold layer using a sputter coater. Fourier infrared characterization of membrane samples using diffuse reflectance conditions, and the wavenumber range was 4000–400 cm^−1^. The high temperature thermogravimetry-differential thermal synchronous analyzer Diamond TG/DTA used a temperature of 30–800 °C, a nitrogen atmosphere with the temperature increase rate of 10° min^−1^. Prior to tests, the membranes were vacuum dried at 70 °C overnight.

### 2.5. Gas Permeation Test

**Figure 1:**
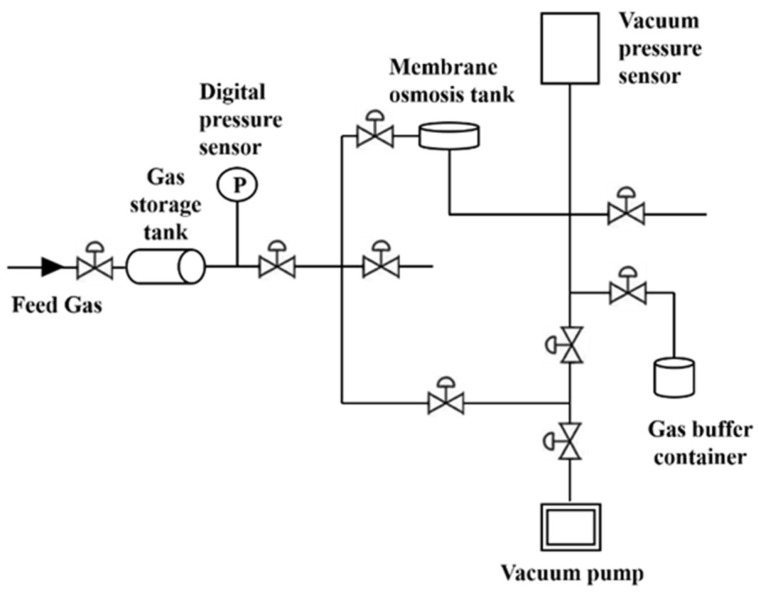
Schematic diagram of a constant volume/variable pressure set-up for the gas permeation measurement.

The gas permeation performance of the mixed matrix membranes was tested by the pure gas constant volume-variable pressure method in the gas permeation in a homemade device, the schematic diagram of the plant is shown in Figure 1. Every membrane was tested three times to confirm the reproducibility of the testing. Before testing, membranes were activated in a vacuum oven at 70 °C for 24 h. Then, the membranes were assembled and placed in an infiltration mold. The gas performance was tested at 25 °C and under 3 bar feed pressure. The definition of permeability is as follows:

(2) $$P=\frac{273.15\times V\times L}{76\times p\times A\times T}*\frac{d_{p}}{d_{t}}*10^{10}$$

where *P* is the permeability of the gas (1 Barrer = 1 × 10^−10^ cm^3^(STP) cm cm^−2^·s^−1^·cmHg^−1^), *V* is the volume of the container to be tested (cm^3^), *L* is the thickness of the membrane (cm), *A* is the effective area of the membrane (cm^2^), *T* is the experimental temperature (K), *p* is the feed pressure (cmHg), and *d_p_*/*d_t_* is the rate of change of pressure over time (cmHg·s^−1^). Another parameter characterizing the gas separation performance of membrane is the ideal selectivity α, which is defined as the ratio of permeability of two gases.

(3) $$\alpha =\frac{P_{i}}{P_{j}}$$

## 3. Results and Discussion

### 3.1. XRD Patterns and SEM Image of MAF-7 Powders

**Figure 2:**
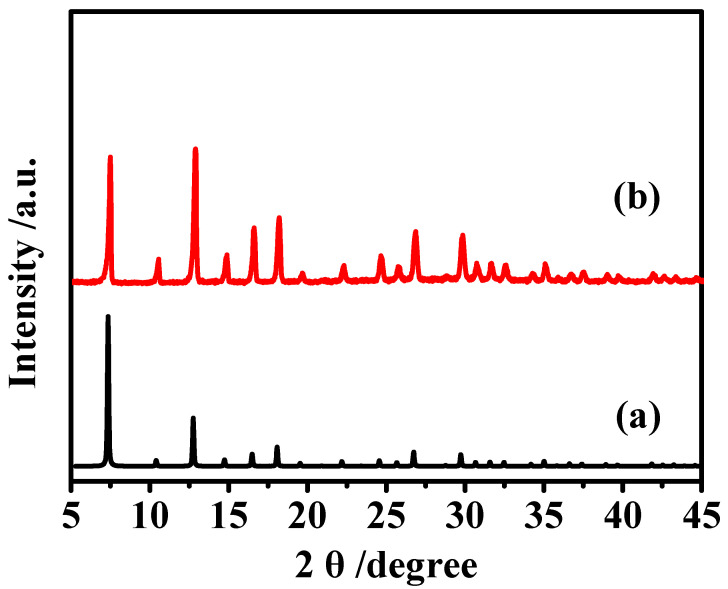
XRD patterns of (**a**) simulated MAF-7 and (**b**) synthesized MAF-7.

**Figure 3:**
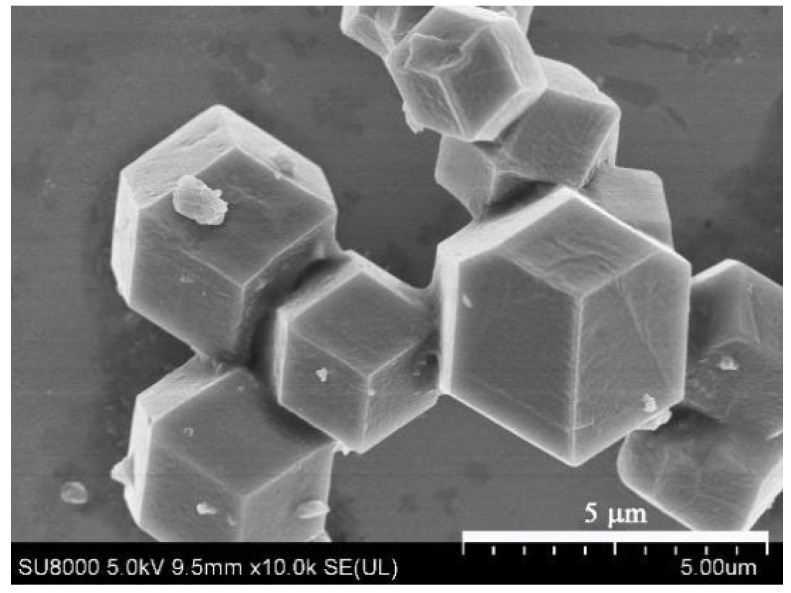
SEM image of MAF-7 powders.

As shown in Figure 2, the characteristic peaks of synthesized MAF-7 at 2θ = 7.3°, 10.3°, 12.7°, 14.7°, 16.5°, 18° are in good agreement with those of the simulated MAF-7. Proving that we have successfully synthesized the pure phase MAF-7 powders (CCDC: 787,579). Figure 3 shows a regular crystal morphology of MAF-7 with the crystals size about 4 µm in average.

### 3.2. XRD characterization of MAF-7/Pebax MMMs

**Figure 4:**
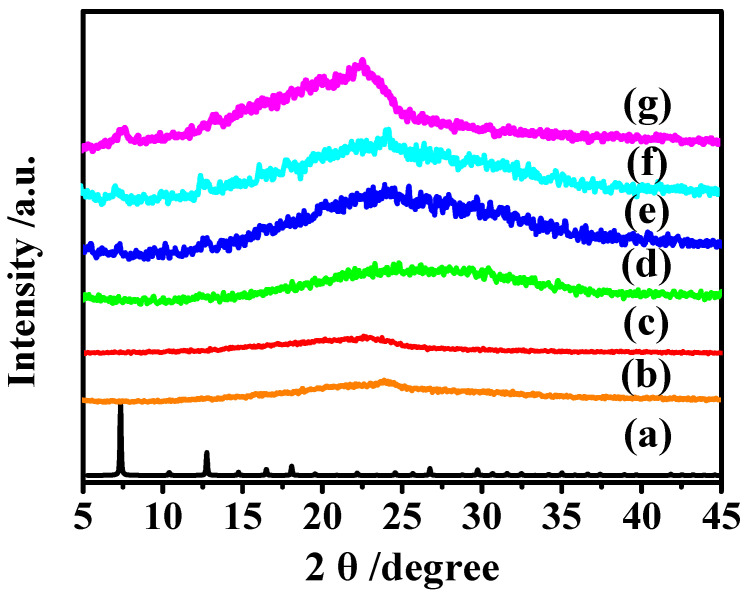
XRD patterns of (**a**) simulated MAF-7, (**b**) P, (**c**) PM 1, (**d**) PM 3, (**e**) PM 5, (**f**) PM 7, and (**g**) PM 9.

The crystal phase of the membranes was analyzed by XRD technique. Pebax-1657 was a block copolymer, with the characteristic peak of crystalline PA appeared at 23.9° [24]. As shown in Figure 4, with a lower filler content (1% and 3%), the characterized MAF-7 peaks did not show obviously in the MMMs, this may be due to a lower filler loading of the membrane with a weak peak intensity. As the loading of MAF-7 increased, the characteristic peak intensities of MAF-7 became more and more obvious. Comparing the simulated crystal structure of MAF-7, we can observe that the characteristic peak of filler at 7.3° in the mixed matrix membrane was gradually enhanced, which indicates that MAF-7 was successfully incorporated into organic phase.

### 3.3. SEM Characterization of Pebax 1657/MAF-7 MMMs

**Figure 5:**
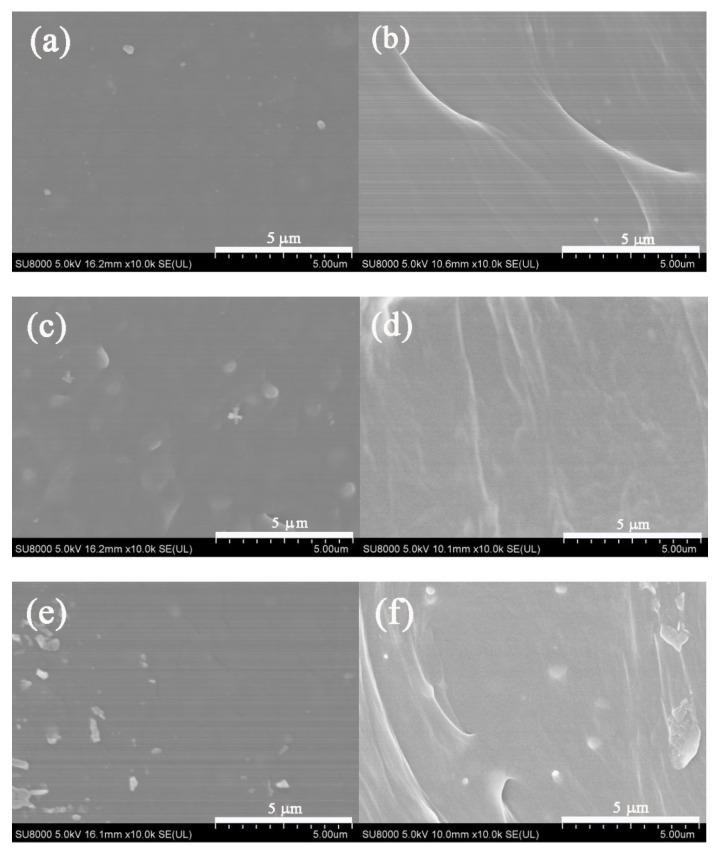

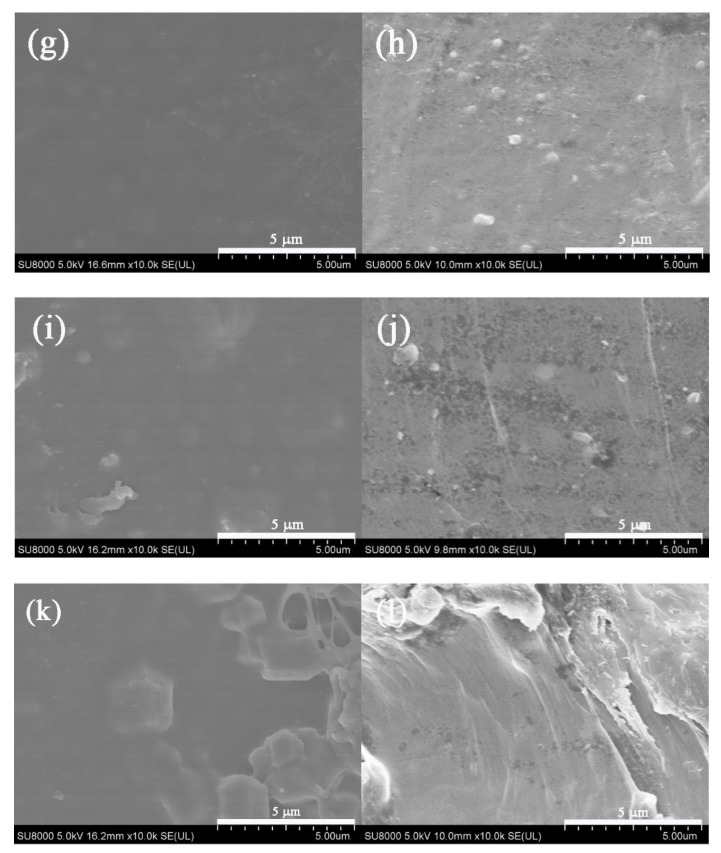
Top-view and cross-section SEM images of (**a**,**b**) P, (**c**,**d**) PM1, (**e**,**f**) PM3, (**g**,**h**) PM5, (**i**,**j**) PM7, and (**k**,**l**) PM9.

The morphologies of prepared Pebax1657/MAF-7 MMMs containing varied loadings of MAF-7 particles were observed as shown in Figure 5. Figure 5a,b demonstrated that the top-view and cross-section morphology of pure Pebax membrane was compact and uniform, and no obvious interface defects were observed. With a lower filler content (from 1% to 5%), the SEM analyses displayed a smooth surface of the obtained MMMs. Due to the low content having good compatibility with the MAF-7 powders in the membrane matrix, no obvious voids and/or cracks were observed in the surface of the Pebax/MAF-7 MMMs, indicating the MAF-7 particles in the membrane matrix have good dispersion and stability. When the filler contents were 7–9 wt.%, the MAF-7 particles did not embed completely in the Pebax matrix. In addition, the self-aggregation of MAF-7 particles was also observed on the membrane surface. When the filler content exceeds a critical loading (7 wt.% here), the particles tend to agglomerate, due to the polymer chains not being able to intimately cover them. In this work, the micrometer size of MAF-7 limits the high loading level [26]. The nonuniform distribution leads to the interfacial defects which is disadvantageous to the gas separation performance of the membrane, as shown in Figure 5i–l.

### 3.4. Thermodynamic Properties of Membranes

**Figure 6:**
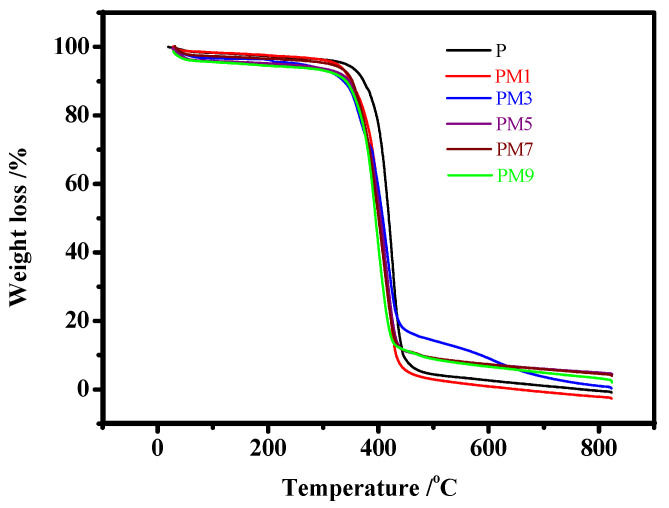
TGA curves of P, PM1, PM3, PM5, PM7, and PM9.

The TGA curves of the pure Pebax membrane and the Pebax 1657 /MAF-7 MMMs with different filler contents are given in Figure 6 to evaluate the thermodynamic properties. The entire thermal decomposition process consists of three major stages of mass loss. The first mass loss stage is about at 100 °C, it ascribes to the evaporation of residual solvent in the membrane; and the deacetylation and depolymerization of Pebax 1657 lead to the second mass loss stage between 100 and 400 °C; for the last mass loss stage, it is due to the residual decomposition of Pebax 1657. This result is consistent with the previous work [32,40]. In detail, with the addition of MAF-7 filler, the initial thermal decomposition temperature of Pebax 1657/MAF-7 MMMs decreased slightly. According to the previous report, the pyrolysis temperature of MAF-7 powder was about 270 °C [38]. Hence, by blending with Pebax and MAF-7 to form a membrane, the final decomposition temperature of Pebax 1657/MAF-7 MMMs is only slightly lower than that of a pristine Pebax membrane. The results demonstrated that the obtained membranes have comparable thermal stability which is sufficient for conventional gas separation applications.

### 3.5. Fourier Transform Infrared Spectroscopy of Membranes

**Figure 7:**
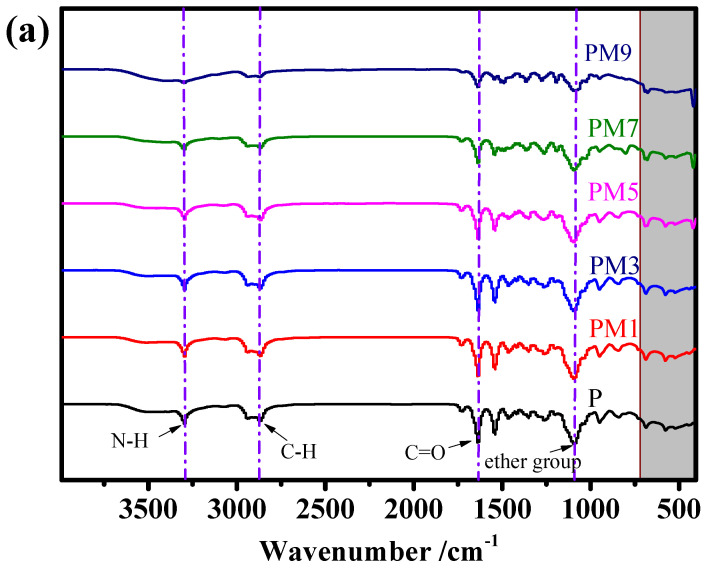

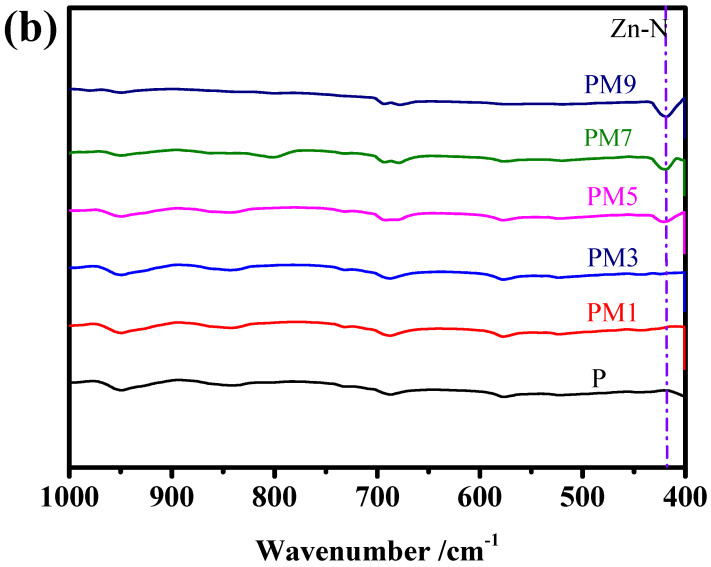
Fourier transform infrared spectroscopy patterns of P, PM1, PM3, PM5, PM7, and PM9. (**a**) 405–3990 cm^−1^ and (**b**) 400–1000 cm^−1^.

FT-IR analysis was conducted to verify the successful blending of Pebax 1657 and MAF-7 crystals. The FT-IR results of pure Pebax-1657 membrane and Pebax-1657/MAF-7 MMMs with varied MAF-7 loadings are shown in Figure 7. As demonstrated in Figure 7a, the characteristic peaks of membranes located at 1091, 1636, 2869, and 3297 cm^−1^ are ascribed to the stretching vibration of the ether group, C=O, C-H, and N-H groups [22,41], respectively. Compared with a pure Pebax membrane, when the loading of filler is 5 wt.%, Pebax 1657/MAF-7 MMMs began to show obvious MAF-7 characteristic peaks in Figure 7b. The characteristic peak at 420 cm^−1^ is attributed to the Zn-N vibration of the mixed matrix membranes, proving we successful blended MAF-7 crystals into the membrane matrix [42,43].

### 3.6. Gas Permeation Performance

#### 3.6.1. Effect of MAF-7 Loading in MMMs

**Figure 8:**
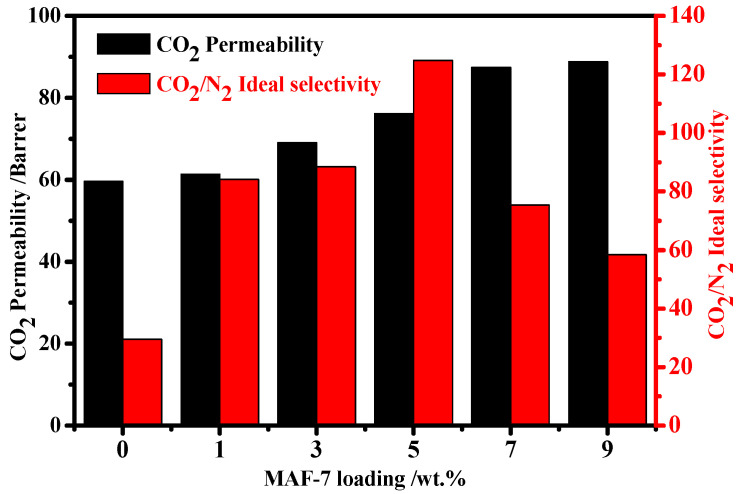
CO_2_ permeability and CO_2_/N_2_ ideal selectivity as a function of different filler loadings.

The pure gas permeation results of the membranes with different filler loading under 3 bar feed pressure and 25 °C are shown in Figure 8. With the increasing content of MAF-7 fillers to 5 wt.%, the CO_2_ permeability and ideal selectivity of Pebax 1657/MAF-7 MMMs increased simultaneously. For dense and nonporous pristine Pebax membrane, the transport of gases through the membrane obeys a solution diffusion mechanism, the PEO section can provide strong affinity with CO_2_, leading to CO_2_ preferentially dissolving in the upstream face of a membrane, diffusing across the membrane, and desorbing from the downstream face of the membrane. Because of the dense membrane layer, the pristine Pebax membrane usually shows a low permeability. As a highly porous framework, MAF-7 has a pore widow of 0.34 nm, which is a suitable size for sieving CO_2_ (0.33 nm) and N_2_ (0.36 nm) [38]. The increasing MAF-7 contents in the membrane matrix could promote the diffusion rate of CO_2_ and have less effect on small sized N_2_, which leads to CO_2_ diffusion being easier than N_2_. On the other hand, the uncoordinated N donor in the MAF-7 three-dimensional framework can adsorb carbon dioxide due to the Lewis acid–Lewis base interaction between the negative charged oxygen atoms of CO_2_ and N-containing organic heterocyclic molecules, which results in an increase in the permeability of the membrane to CO_2_ and the ideal CO_2_/N_2_ selectivity [44]. When the filler loading is 5 wt.%, the CO_2_ permeability is 76.15 Barrer, and the CO_2_/N_2_ ideal selectivity reaches the highest value of 124.84. Compared with the pure membrane, the ideal selectivity of CO_2_ permeability and CO_2_/N_2_ of Pebax 1657/MAF-7 MMMs has been increased by 27.75% and 323.04%, respectively. Afterwards, as the MAF-7 filler continues to increase, the fillers tend to agglomerate, and the membrane has more interface defects, resulting in a decrease in CO_2_/N_2_ selectivity. However, even with the filler loading increase to 9 wt.%, the CO_2_/N_2_ ideal selectivity of MMMs is still higher than that of the pure Pebax membrane. Obviously, the introduction of MAF-7 filler in Pebax matrix can improve the gas separation performance and the results also show the great potential of MAF-7 filler-based membrane in the field of gas separation.

#### 3.6.2. Effect of Feed Pressure

**Figure 9:**
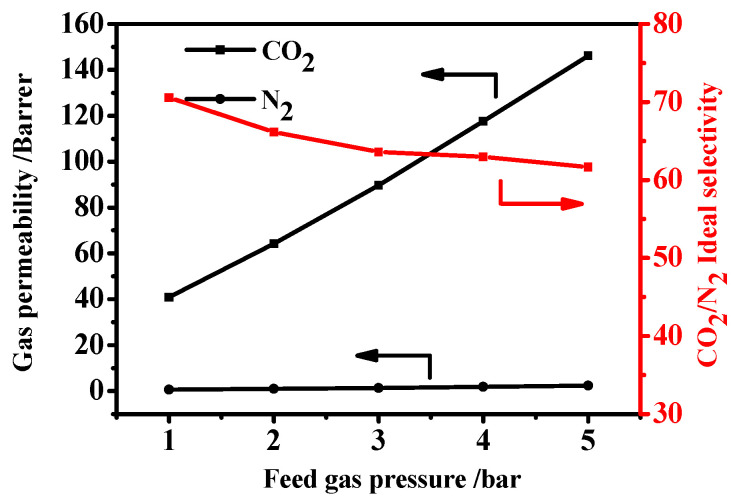
Gas separation performance of PM5 MMM under different feed pressure.

Figure 9 illustrates the relationship between feed pressure and separation performance on membranes with 5 wt.% MAF-7 loading. The gas permeability of CO_2_ and N_2_ also showed the regularity of increasing as the feed pressure increased, but the CO_2_/N_2_ selectivity showed the opposite changing regularity. The separation principle of the rubbery polymer membrane was followed by the solution-diffusion mechanism. When the pressure was increased, the membrane increased the diffusion rate and solubility of the gas, resulting in an increase in the permeability of both gases [45]. However, the N_2_ molecules were less affected than CO_2_ molecules with increasing feed pressure, resulting in a decrease in selectivity. The phenomenon of the increasing feed pressure was consistent with previously studies [46].

#### 3.6.3. Effect of Testing Temperatures

**Figure 10:**
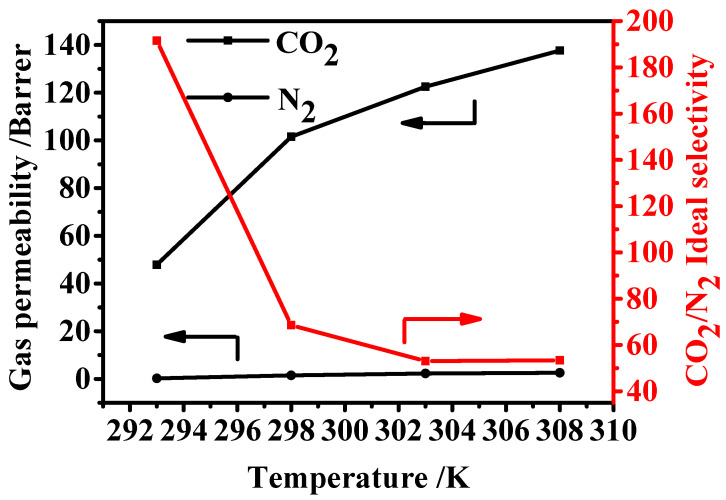
Gas separation performance of PM5 MMM at different testing temperatures.

Figure 10 illustrates the influence of the operating temperatures on the separation performance of the membrane. As the testing temperature increased, the gas permeability increased, and the selectivity showed the same variation trend. Generally, the gas diffuses among the MMM layer through the porous and free volumes in the membrane matrix, and the free volumes are randomly formed by the motions of the organic chains. The increased testing temperature induced the increased flexibility of the polymer chains, leading to more free volumes of the membrane and faster diffusion of the gas [47]. Thus, the increasing permeability of CO_2_ and N_2_ was mainly due to the increased diffusion of gas molecules at high temperature, and the increased flexibility of the membrane matrix [48]. However, at high testing temperature, the membrane showed a low affinity for gases, especially for CO_2_. That is not conducive to promoting separation through preferential adsorption on the membrane. Obviously, as the temperature rises, it resulted in a decrease in CO_2_/N_2_ selectivity [47,49]. A similar result was also observed in MOF-801 mixed matrix membranes [32].

The results indicate that the gas permeability through the MMMs is a temperature activation process; thus, the activated energies of the gas permeability can be obtained by correlation with the Arrhenius equation:

(4) $$P=P_{0}\exp(-\frac{E_{p}}{RT})$$

where *P* is the permeability coefficient of the corresponding gas (Barrer), *P*_0_ is the pre-exponential factor (Barrer), *Ep* is the permeation activation energy (kJ mol^−1^), *R* is the gas universal constant (J mol^−1^K^−1^), and *T* is the absolute temperature (K). By linear fitting and regressing, the *E_p_* of CO_2_ is 51.05 ± 2.03 kJ mol^−1^, which is significantly lower than that of N_2_ (114.32 ± 5.16 kJ mol^−1^), indicating that CO_2_ permeability is less sensitive to the temperature change than N_2_ permeability, because CO_2_ has a smaller kinetic diameter, and a larger diffusion coefficient through membrane than that of N_2_. Hence, with an increase in the testing temperature, the increase in both CO_2_ and N_2_ permeability led to a decrease in CO_2_/N_2_ selectivity.

### 3.7. Comparison with Previous Research

The gas permeation performance of the synthesized Pebax 1657/MAF-7 MMMs was compared with the Pebax-based mixed matrix membranes in previous publications. As shown in Table 2, Deng et al. reported a Pebax/ZIF-C (zeolitic imidazolate framework cuboid) membrane, which showed high CO_2_ permeability accompanied with a CO_2_/N_2_ selectivity of 47.1 [50]. Liu et al. published about COF-5 as fillers to fabricate MMMs that exhibited an enhanced CO_2_ permeability of 493 Barrer together with a CO_2_/N_2_ selectivity of 49.3 [51]. The above research followed a trade-off effect between permeability and selectivity. In comparison, the compactness of the membrane which is represented by selectivity is critical for high quality membrane. Therefore, it is desired to fabricate membranes possessing high selectivity and comparable permeability. We used a simple and feasible casting method by incorporating MAF-7 crystals into Pebax membrane to construct a high-performance membrane which exhibited improvements in CO_2_ permeability with remarkable elevated CO_2_/N_2_ selectivity of 124.84.

**Figure 11:**
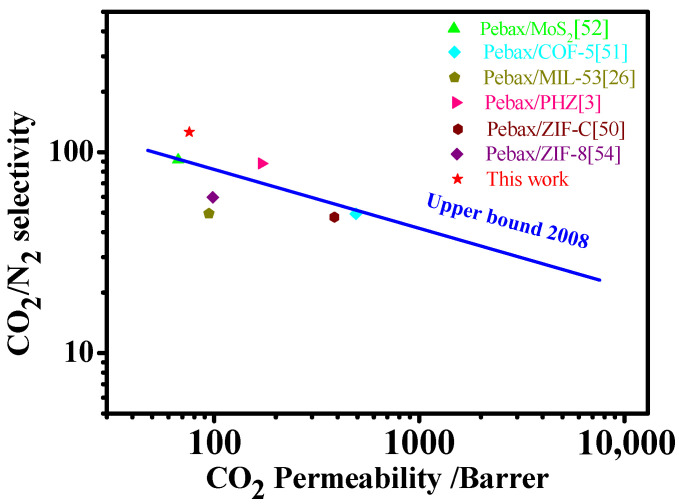
Comparison of Pebax 1657/MAF-7 MMMs with the Robeson upper bound in 2008.

Finally, the gas separation performance of the prepared Pebax 1657/MAF-7 mixed matrix membrane was compared with the Robeson upper bound in 2008, and the results are shown in Figure 11. Compared with some Pebax-based mixed matrix membranes, the Pebax 1657/MAF-7 mixed matrix membrane has better selectivity. After the addition of MAF-7 filler, the gas separation performance of the prepared Pebax 1657/MAF-7 MMMs is higher than that of pure Pebax membrane. The separation performance reaches the highest when the filler content is 5 wt.%, which has exceeded the Robeson upper bound in 2008. The results show that Pebax 1657/MAF-7 mixed matrix membrane is an alternative membrane material with potential advantages in the field of CO_2_ separation and capture.

## 4. Conclusions

In summary, Pebax 1657/MAF-7 MMMs were successfully prepared by blending with Pebax 1657 and MAF-7 fillers in this work. As a result of the organic ligands in the MAF-7 structure, the MAF-7 crystals showed good compatibility with Pebax polymers, combining with the uncoordinated N donor in the MAF-7 crystals, the obtained membrane showed an increasing ideal selectivity of CO_2_/N_2_. The permeance results showed that Pebax 1657/MAF-7 MMMs have a better pure gas permeability performance with the filler content of 5 wt.%; CO_2_ permeability reached 76.15 Barrer with a CO_2_/N_2_ ideal selectivity of 124.84, which exceeds the Robeson upper bound in 2008. The obtained membrane showed 323.04% enhancement in selectivity of CO_2_/N_2_ and 27.74% increase in permeability of CO_2_ compared to the pristine membrane at 25 °C and 3 bar. Compared with the complicated chemical methods to modify fillers for improving the chemical affinity toward guest molecules and the compatibility between fillers and Pebax, MAF-7 (other MAFs as well) crystals may be ideal candidate fillers to fabricate high performance membrane for CO_2_/N_2_ separation in practical applications.

## Figures and Tables

**Table 1:** Membranes fabricated in this work.

Membrane	Abbreviation
Pebax 1657	P
Pebax 1657/MAF-7-1	PM1
Pebax 1657/MAF-7-3	PM3
Pebax 1657/MAF-7-5	PM5
Pebax 1657/MAF-7-7	PM7
Pebax 1657/MAF-7-9	PM9

**Table 2:** Comparison of Pebax 1657/MAF-7 MMMs with previous publications.

Membrane	P_CO2_	CO_2_/N_2_	Refs.
	(Barrer)	Selectivity	
Pebax/MoS_2_	67	91	[52]
Pebax/NIPAM-CNTs	87	53	[21]
Pebax/FS7	60.15	91.14	[53]
Pebax/COF-5	493	49.3	[51]
Pebax/MIL-53	95.7	49.9	[26]
Pebax/ZIF-8	99.7	59.6	[54]
Pebax/PHZ	172.4	87.9	[3]
Pebax/ZIF-C	387.2	47.1	[50]
Pebax/ZCN	110.51	84.35	[45]
Pebax/ZnO@ZIF-8 HNTs	140	67	[55]
Pebax/GO	100	91	[56]
**Pebax 1657/MAF-7**	**76.15**	**124.84**	**This work**

## Data Availability

The data presented in this study are available on request from the corresponding author.
